# High Level of SARS-CoV-2 Infection in Young Population Is a Predictor for Peak Incidence

**DOI:** 10.3389/fmicb.2022.891646

**Published:** 2022-05-31

**Authors:** Haeyoun Choi, Sun Shin, Seung-Jin Hong, Sang-Uk Seo, Mun-Gan Rhyu

**Affiliations:** Department of Microbiology, College of Medicine, The Catholic University of Korea, Seoul, South Korea

**Keywords:** SARS-CoV-2, COVID-19, true infection, age groups, morbidity, epidemiology

## Abstract

South Korea adopted stringent preventive measures against Coronavirus virus disease 2019, resulting in three small and one large outbreaks until January 15, 2022. The fatality rate was 2.5-fold higher during peak transmission periods than in base periods. As new variants of severe acute respiratory syndrome Coronavirus 2 (SARS-CoV-2) are continuously emerging, the need for understanding their epidemic potential remains necessary. In South Korea, the epidemiologic data obtained from mass diagnostic testing enabled investigation of the true number of infected cases, exact incidence, and fatality numbers. Analysis found a similarity between estimated infection rates and confirmed cases. This suggested that the number of confirmed cases had an influence on the fatality rate as a quantitative parameter. The fatality rate decreased even as infection with SARS-CoV-2 variants rose. In comparative analysis, the confirmed cases in young people (ages 20–29) increased prior to every outbreak peak and marked the tipping point in infection spread. These results indicate that a high level of SARS-CoV-2 infection in young population drives peak incidence and mortality across all age groups.

## Introduction

Coronavirus (CoV) is divided into four different genera that can be found in a broad spectrum of animal hosts (Woo et al., 2012). Since the severe acute respiratory syndrome (SARS)-CoV was first reported in 2002, two additional human CoVs (NL63 and HKU1) were isolated but the majority of newly discovered CoVs were found in animals (Fouchier et al., 2004; Woo et al., 2005). Humans may not be the optimal host for maintaining the virulence and prolonged infection, because few CoVs transcend interspecies barriers to infect humans. Indeed, SARS-CoV and Middle East respiratory syndrome (MERS)-CoV epidemics did not last more than 9 months or became sporadically endemic despite having exhibited significant morbidity and mortality worldwide (Farrag et al., 2021). Unlike MERS-CoV and SARS-CoV infections, CoV disease 2019 (COVID-19) caused by SARS-CoV-2 has continued to spread for more than 2 years. SARS-CoV-2, like other RNA viruses, is prone to mutates and has generated multiple variant clades. Each variant has distinct epidemiologic characteristics, and their backgrounds remain obscure.

The population viral load (PVL) covering all apparent and inapparent patients has been theoretically presented as a standard measure of the infection extent and spread of human immunodeficiency virus (HIV) [National Center for HIV/AIDS, Viral Hepatitis, STD, and TB Prevention (U.S.). Division of HIV/AIDS Prevention, 2011]. The quantification of infected viral particles is useful for predicting infection incidence and severity in a community, however, both total infections and PVL cannot be accurately estimated for SARS-CoV-2 due to the high proportion of asymptomatic patients and rapid viral transmission. One study reported a reverse correlation between onset age and asymptomatic rate, making disease trend analysis difficult for the entire population (Sah et al., 2021). Case fatality rate (CFR), the ratio of deaths to reported cases, has been calculated without considering total infections in the general population. This observed mortality of SARS-CoV-2 varies over time due to the heterogeneity of infection-tracing and -testing capacity among countries. Also, the mortality rate of SARS-CoV-2 infection increases in an age-dependent manner (O’driscoll et al., 2021). The infection fatality rate (IFR) is calculated as the ratio of deaths to the true number of total infections, providing the exact fatality rate. Therefore, the number of confirmed cases and IFR are necessary to precisely track the progress of the SARS-CoV-2 pandemic as well as for understanding variant traits.

Unlike many countries, South Korea implemented extensive diagnostic testing for SARS-CoV-2, which covered most people who had close contact with confirmed cases (Shin et al., 2021). In South Korea, the number of diagnostic tests per confirmed case from March 3, 2020 to January 1, 2022 was 135, 7.6 times higher than in the United States (Our World in Data, 2022). In addition, South Korea adopted testing-tracing-treatment (so-called 3T) strategy and strict social distancing to minimize baseline-level outbreaks (Park et al., 2020). Consequently, we could precisely determine the IFR of SARS-CoV-2 infections in South Korea based on the quantitative degree of viral infection for a given period of time.

Here, we aimed to precisely present the progress of SARS-CoV-2 infection affected by the number of cases during the peak and base periods of the epidemics. We analyzed the number of weekly confirmed cases and deaths from data from January 20, 2020 to January 15, 2022, a period that spanned the Delta pandemic. The incidence of SARS-CoV-2 infection in a young population (ages 20–29 years) increased with the appearance of new variants and their infections became mild. The high incidence in the young population was followed by increased incidence and fatalities in the total population. Using the epidemiologic data, we calculated the IFR of COVID-19 in the total population as well as in 10-year age groups.

## Materials and Methods

### Data Source

Daily SARS-CoV-2 incidence and mortality counts in South Korea were collected from the official COVID-19 website of the Korea Disease Control and Prevention Agency (KDCA).^1^ Since the first confirmed SARS-CoV-2 infection in South Korea in January 2020, 688,000 cumulative confirmed cases and 6,281 deaths were reported as of January 15, 2022. Data by ages of the confirmed cases and deaths due to SARS-CoV-2 are available for the following age groups, which are shown in years: 0–9, 10–19, 20–29, 30–39, 40–49, 50–59, 60–69, 70–79, and ≥80. Data were summarized for each week to reduce the potential week-related fluctuations. Age-specific changes in the number of confirmed cases during a short-term outbreak were calculated daily.

### Variant Data

Lineages of SARS-CoV-2 identified in South Korea were obtained by downloading “metadata” from GISAID on December 16, 2021 for all 20,233 complete viral genome sequences sampled from South Korea since January 2020. These metadata have Pango lineages (Rambaut et al., 2020) v.3.1.17 (2021-12-06 released) assigned to each genome sequence. The data were summarized monthly by sample dates and Pango lineage category.

### Infection Risk Adjusted for Population Ratio

In our analyses, we defined *C*_*a*_,*_*t*_* as the number of confirmed cases for age group *a* ∈ *A* in a calendar week or outbreak period (*t*), and *C*_*t*_ = $\sum_{a\,\in \,A\,}C_{a,t}\,$ as the total number of confirmed cases in a week or outbreak period (*t*). *I*_*a*_,*_*t*_* and *I*_*t*_ were the number of true infections and *D*_*a*_,*_*t*_* and *D*_*t*_ were the number of deaths for age group *a* and week or outbreak period *t*. In order to understand the characteristics of age-specific SARS-CoV-2 infection, the risk ratio (*R*) was calculated by dividing the proportion of age-specific confirmed cases *C*_*a*_ by the proportion of the age group population as follows:


$$R_{a}=\frac{C_{a}/C_{t}}{P_{a}/P},$$

where *P*_*a*_ denotes the population numbers in age group *a* ∈ *A* and *P* is the total population of South Korea, *P* = ∑_*a*_ ∈ *A P*_*a*_.

### Fatality Rate

We defined the observed CFR*_*a,t*_* as the number of deaths (*D*_*a*_,*_*t*_*) divided by the number of confirmed cases *C*_*a*_,*_*t*_*. The IFR was based on the true number of infections. As the number of true infections (*I*) is unknown, the effective IFR was estimated by taking into account the age-specific IFR estimate based on seropositive data of 34 geographic locations, including South Korea as follows (Levin et al., 2020):


$${\hat{\text{IFR}}}_{a}=10^{-3.27+0.0524\times a},$$

where the standard errors for estimated coefficients are 0.07 and 0.0013, respectively.

With the assumption that the age distribution influences the number of true infections, the effective IFR can be calculated as follows by a weighted average of age-specific IFR estimates,


$${\hat{\text{IFR}}}_{\text{eff}}=\sum_{a\,}\in \,A\,{\hat{w}}_{a}\times {\hat{\text{IFR}}}_{a},$$

where ${\hat{w}}_{a}$ is an estimator for the fraction of infections *I*_*a*_/*I* in age group *a*. The number of true infections *I*_*a*_ is estimated by considering the age-dependent factor *f via* this equation:


$${\hat{I}}_{a}={\hat{f}}_{a}\times C_{a}=\frac{{\text{CFR}}_{a}}{{\hat{\text{IFR}}}_{a}}\times C_{a}=\frac{\frac{D_{a}}{C_{a}}}{{\hat{\text{IFR}}}_{a}}\times C_{a}=\frac{D_{a}}{{\hat{\text{IFR}}}_{a}},$$

where ${\hat{f}}_{a}$ denotes the estimated factor in age group *a*.

Due to the small number of confirmed deaths in younger age groups, ${\hat{I}}_{a}$ was calculated for ages ≥50. Considering the period from confirmation of infection to death, for confirmed cases in the *I*_*a*_ calculation, we used data obtained by December 31, 2021, and data for COVID-19 deaths until January 15, 2022.

## Results

### Epidemiologic Characteristics of Peak and Base Periods of SARS-CoV-2 in South Korea

**FIGURE 1:**
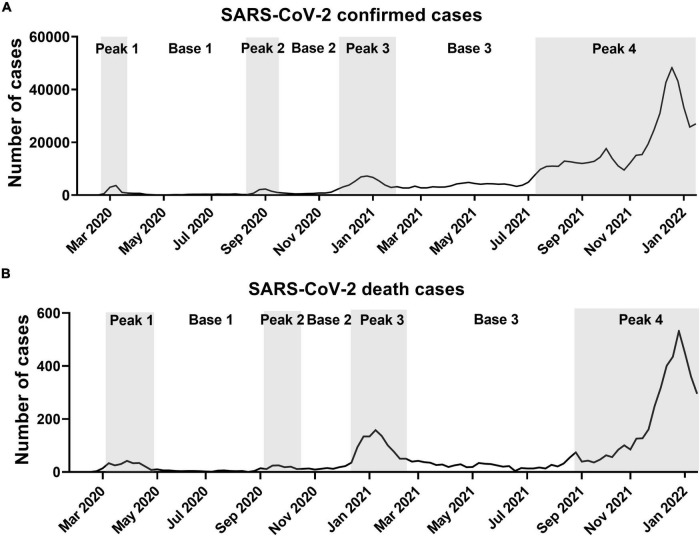
Weekly confirmed cases and deaths due to SARS-CoV-2 in South Korea. **(A)** Weekly confirmed cases. **(B)** Weekly death cases. Data are from start of epidemic until January 15, 2022. Peak periods are shown in gray.

During the SARS-CoV-2 pandemic, the shape of the incidence curve generated by plotting the number of weekly confirmed cases revealed four peak periods and interleaved three base periods (Figure 1A). The number of deaths also produced four peaks 2–3 weeks later than the confirmed cases (Figure 1B). The fourth peak is still in progress. The starting point of a peak period was defined by the doubling of weekly confirmed cases or deaths over 1 week. The ending point was defined by the time point at twice the number of weeks taken from the starting point to the peak. The duration of the first three peaks (5–11 weeks) was shorter than those of the three bases (9–22 weeks). The average number of weekly confirmed cases in peak periods was 5.6 ± 0.7 times higher than in base periods. For death, the average number of confirmed deaths in peak periods was 6.0 ± 1.7 times higher than that in base periods. These repetitive peak and base periods were closely related to changes in fatalities. Overall, as shown in Table 1, the height and duration of peaks of confirmed cases and deaths grew taller and longer over time. The number of confirmed cases in base 3 remained at elevated levels compared to the previous two base periods. The heights and lengths of peak 4 were much higher to the previous three small peaks.

**TABLE 1:** Demographics of SARS-CoV-2 by specified periods.

	Peak 1	Base 1	Peak 2	Base 2	Peak 3	Base 3	Peak 4
**Confirmed cases**							
Total cases	8,867	5,701	7,577	6,369	50,270	81,497	528,669
Average cases/week	1,773	285	1,515	708	4,570	3,704	18,881
Maximum cases/week	3,608	–	2,300	–	7,206	–	48,276
Minimum cases/week	–	65	–	480	–	2,673	
Duration of peak (in weeks)	5	20	5	9	11	22	28
Time period	Feb 2020–Mar 2020	Mar 2020–Aug 2020	Aug 2020–Sep 2020	Sep 2020–Nov 2020	Nov 2020–Jan 2021	Feb 2021–Jul 2021	Jul 2021–Jan 2022
**Death**							
Total deaths	240	69	123	73	1,048	563	4,166
Average deaths/week	27	4	18	12	81	24	181
Maximum deaths/week	42	–	25	–	158	–	532
Minimum deaths/week	–	0	–	9	–	4	–
Duration of peak (in weeks)	9	17	7	6	13	24	23
Time period	Mar 2020–Apr 2020	May 2020–Aug 2020	Aug 2020–Oct 2020	Oct 2020–Nov 2020	Nov 2020–Feb 2021	Feb 2021–Aug 2021	Aug 2021–Jan 2022
							

### The Appearance of SARS-CoV-2 Variants in South Korea

**FIGURE 2:**
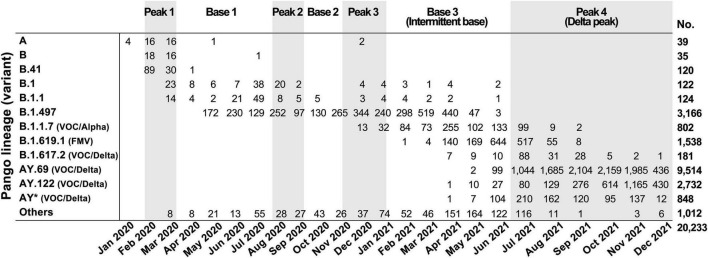
SARS-CoV-2 variants identified in South Korea by month. Variants were classified based on Pango lineage v.3.1.17; WHO classifications are in parentheses. VOC, variant of concern; FMV, formerly monitored variant; AY*, AY lineages excluding AY.69 and AY.122.

Consistent with the epidemiological data, variants found in South Korea remained largely unchanged until Delta variants started to dominate in peak 4 (Figure 2). The predominant lineages were B.41 for the peak 1 period, B.1.497 for the peak 2 and 3 periods, and AY.69 for the peak 4 period. Lineage B.1.497, which led to SARS-CoV-2 infection for the longest period in South Korea, was the dominant subgroup from May 2020 to April 2021 across the entire peak 2, peak 3, and part of base 3. Even when B.1.1.7 rapidly spread worldwide in late 2020 (Alpert et al., 2021; Kraemer et al., 2021), B.1.497 and B.1.619.1 variants outnumbered B.1.1.7 in South Korea. However, as the infection progressed from peak 3 to base 3 periods (from March to May 2021), the proportion of the B.1.497 variant gradually decreased from 80 to 9%. Of note, during this period several World Health Organization (WHO)-designated variants of concern (VOC) were reclassified (World Health Organization, 2021). Among these, Alpha B.1.1.7 and formerly monitored variant (FMV) B.1.619.1 emerged sequentially. Upon reaching peak 4 in July 2021, the VOC Delta (B.1.617.2 and all descendent lineages) quickly dominated and occupied up to 94% of peak 4. Accordingly, base 3 is hereinafter referred to as intermittent base and peak 4 to Delta peak.

### Analysis of Fatality Rate Based on Peak and Base Periods Associated With the Number of Confirmed Cases

We verified whether the number of confirmed cases functioned as an epidemic parameter standing for true infection cases. For this purpose, the similarity between reported confirmed cases and true infection cases was mathematically calculated as follows:


$$I_{a}=\frac{D_{a}}{\hat{I\,F\,R_{a}}}=\frac{D_{a}}{10^{-3.27+0.0524\times a}}$$

where *I*_*a*_ is true infection cases, *D*_α_ is number of deaths, and *IFR*_α_ is infection fatality rate for age group.

**FIGURE 3:**
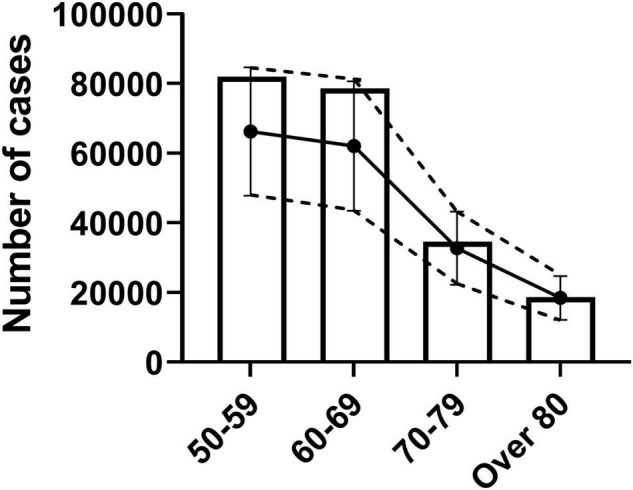
Verification of confirmed cases as epidemic parameter for true infection cases. Analysis of similarity between reported confirmed cases and age-specific estimated true infection cases. Estimated true infection cases with standard error are shown as dotted lines with error bars. Bar graph shows the reported confirmed cases by age group.

The reported confirmed cases in each age groups were within 1 standard error of the estimated confirmed cases (Figure 3). Specifically, estimated cases were 18,421, 32,707, 62,074, and 66,217 while reported confirmed cases were 20,655, 38,669, 90,604, and 92,341 for age groups of over 80, 70–79, 60–69, and 50–59, respectively.

**FIGURE 4:**
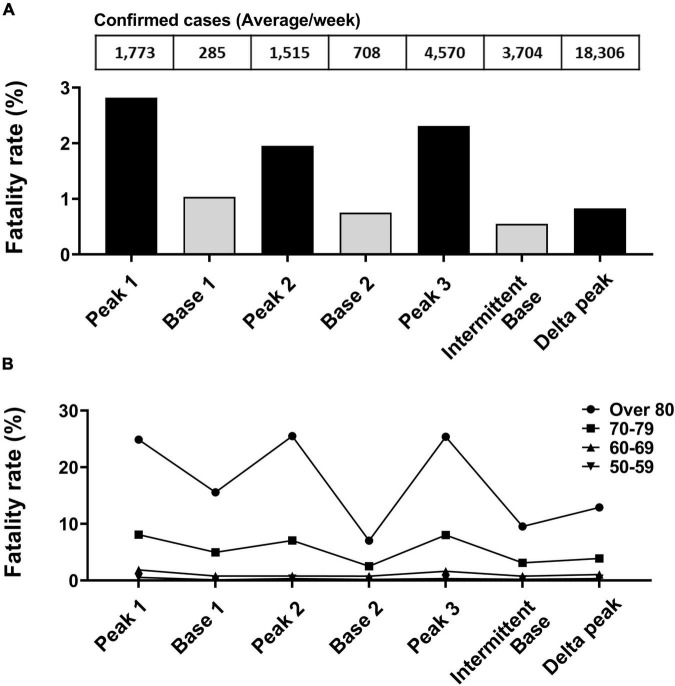
Fatality rate analysis based on peak and base periods. **(A)** Deaths due to SARS-CoV-2 divided by number of confirmed cases for each specified period. **(B)** Fatality rate change by age group between periods.

The fatality rate was calculated by dividing deaths by the number of confirmed cases of each peak or base period (Figure 4A). The fatality rate during peak periods was higher (range, 0.83–2.82%) than during base periods (range, 0.55–1.03%). The fatality rate of the smallest peak (1.95%, peak 2) was the lowest among the three small peaks. In analyses of age groups, the fatality rate was highest in the oldest group and followed sequentially by persons in their 70s, 60s, and 50s. In all age groups, the number of confirmed cases was associated with the fatality rate (Figure 4B).

After the peak 3 period and the appearance of Alpha and Delta variants, the fatality rate remained low despite more confirmed cases than recorded in prior peak and base periods.

### SARS-CoV-2 Variants Shifted the Main Age of Infection

**FIGURE 5:**
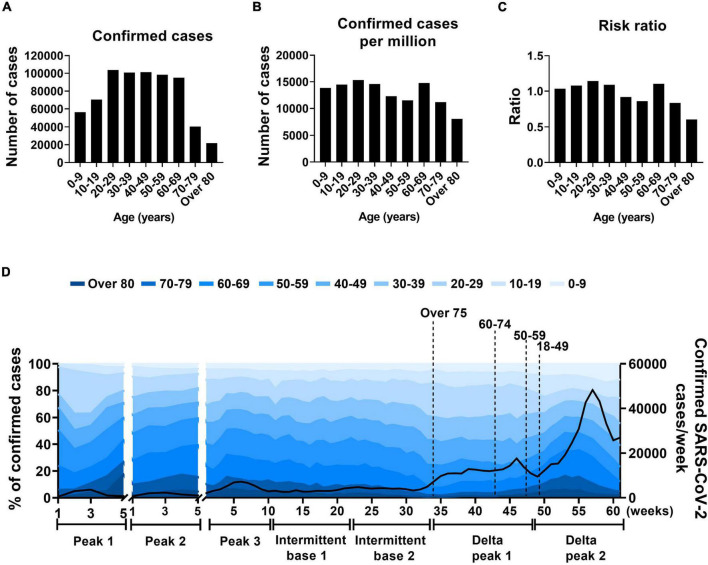
Age distribution in SARS-CoV-2 infection. **(A–C)** Total confirmed cases of SARS-CoV-2, confirmed SARS-CoV-2 cases per million populations, and risk ratios by 10-year age group. **(D)** Proportions of SARS-CoV-2 cases by total confirmed cases. Solid line indicates weekly confirmed cases, x-axis indicates number of weeks of specified periods, and dotted lines indicate when vaccinations reached 80% for each age group.

During the study period, persons in their 20s had the highest number of confirmed cases, 103,708 (Figure 5A). When confirmed cases were compared with South Korean demographic groups, persons in their 20s and 60s had the highest number of confirmed cases per 1 million population (15,302 and 14,772, respectively). Those two age groups accounted for the highest risk ratios among young- and old-age groups: 1.14 and 1.10, respectively (Figures 5B,C). When sex-specific difference in fatality rate was analyzed, no clear difference was seen with fatality rate of 0.90% for men and 0.92% for women (Supplementary Figure 1).

To further analyze the age distribution of SARS-CoV-2 infections, the intermittent base (base 3) was divided into intermittent bases 1 and 2 based on the shift of the major variant (Figure 5D). The Delta peak was also divided into Delta peaks 1 and 2 based on the number of weekly total confirmed cases in which Delta peak 2 showed a steep increase 16 weeks after Delta peak 1 (Figure 5D). During the peak 2 and 3 periods, the proportion of confirmed cases was lower in the 20s age group than in the 60s age group (11.5% and 12.7% [peak 2] and 20.1% and 15.9% [peak 3], respectively). The confirmed cases in the age 20s group gradually increased and in the age 60s group decreased during the intermittent base period (22 weeks) with no vaccine effect. The confirmed cases of the two age groups (20s and 60s) reached maximum and minimum proportions (22.1 and 8.3%, respectively) in the intermittent base 2 period. Vaccination program in South Korea started from February 2021. Vaccination was prioritized to front line health care workers, long term care residents and the people over 75 years of age. By October 2021, when Delta peak 1 was over, the overall proportion of fully vaccinated population reached 80% (Supplementary Figure 2). Vaccinations of the 20s and 60s groups were presumed to have a preventive effect in the Delta peaks 2 and 1, respectively (Figure 5D).

### SARS-CoV-2 Infection of the Young Population Marked the Tipping Point in the Pre-peak Period

**FIGURE 6:**
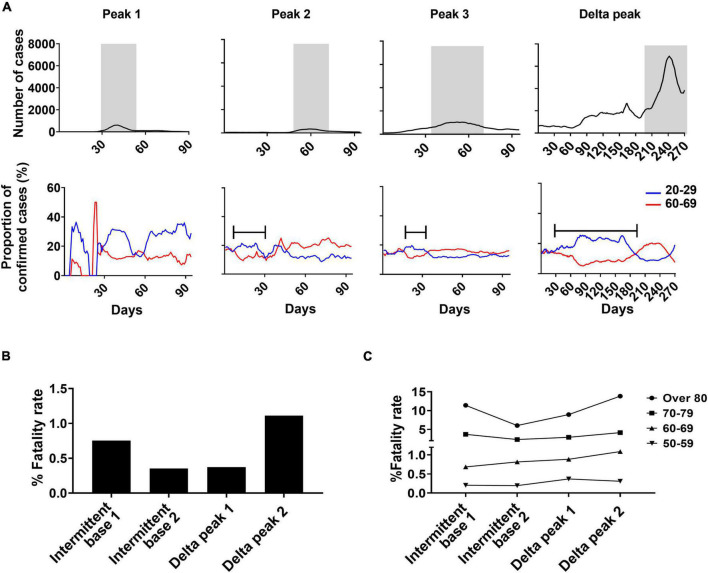
Analysis of age shifts in primary SARS-CoV-2 infections and fatality rates. **(A)** Changing patterns of proportions of confirmed cases in the 20–29 and 60–69 years-old age groups during each peak in relationship to weekly confirmed cases of SARS-CoV-2. Gray indicates peak periods of confirmed cases. Bars indicate pre-peak period when the proportion of confirmed cases increased to 20% in Peaks 2 and 3 and 25% in the Delta peak. Total fatality rate **(B)** and fatality rate by age group **(C)** for specified periods.

Of the two main infection age groups, those in their 20s tended to have more confirmed cases in a pre-peak period and those in their 60s during a peak period (Figure 6A). The confirmed cases of the 20s group increased to 20% on the brink of the peak period. The pre-peak durations with sustained higher proportions of confirmed cases of the 20s age group were similar when measured at 40 days and 17 days before peaks 2 and 3, respectively. After the pre-peak period, the total confirmed cases in total population rapidly increased in accordance with the increase of confirmed cases in the 60s group (Figure 6A). During the Delta peak 1 period, the confirmed cases of the 20s and 60s age groups, respectively, were of high (23%) and low (8%) proportions that were sustained for 187 days. In the 60s age group, confirmed cases increased during the Delta peak 2 period. Overall, the number of confirmed cases in the 20s age group increased repeatedly in the pre-peak periods of both small- and large-peak outbreaks, even though those in their 20s were infected for a longer period in Delta peak (16 weeks).

The Delta variant infection period had low-level mortality despite the increased number of total confirmed cases (Figure 6B). Of the two Delta peaks, Delta peak 2 resulted in increased mortality in those 60 and older compared to Delta peak 1. The fatality rate of the 50s age group slightly declined in the Delta peak 2 period because of the vaccine effect (Figure 6C).

## Discussion

In a previous study, the effective IFR of SARS-CoV-2 was estimated by the age-specific incidence based on seropositive data (Levin et al., 2020). The number of confirmed cases reported by South Korea was within 1 standard error of the estimated infection cases based on seropositive data (Figure 3). Thus, the nation-wide screening was found to cover enough potential cases in the total population to determine the true number of SARS-CoV-2 infected individuals in South Korea. The short-term fatality rate was accurately evaluated by matching the peak periods of confirmed infections and deaths (Figure 4). Throughout the pandemic, there were high fatality rates during peak periods and low fatality rates during base periods. Increase in fatality rate during peak periods compared to base periods could be explained by the increase of infection among old age group which account for most of the death cases. Such temporal and quantitative links between incidence and mortality represented the level of infection in the population with no biased diagnoses. These findings for acute SARS-CoV-2 infections indicate that stringent preventive measures are of greatest importance in lowering the fatality rate by lowering the number of confirmed cases.

As a rule, the incidence of SARS-CoV-2 infection is higher in younger populations than in older populations (Stringhini et al., 2020; Takita et al., 2020). The initial outbreaks are thought to have started in a young age group and spread to an older group (Boehmer et al., 2020; Lau et al., 2020; Oster et al., 2020; Monod et al., 2021; Tran Kiem et al., 2021). In the early stages of an outbreak, the incidence rate tends to be underestimated due to a lack of epidemiological information. In this study, people in their 20s and 60s were found to serve as the two driving groups in SARS-CoV-2 infection. The number of confirmed cases in the younger age group tended to increase repeatedly during pre-peak periods that were close to the peak periods except for the first peak period. When we evaluated the data for South Korea, we found that a new wave of SARS-CoV-2 infections could be predicted by monitoring age-specific infection cases. Pre-peak increases in confirmed cases were obvious in the 20s age group. The increase in infections in persons aged 20–29 before the pre-peak tipping point was a warning about an impending peak outbreak. As also reported in a number of studies, increase of infection in young aged group who are more socially active leads to the spread of infection in other age groups (Stringhini et al., 2020; Monod et al., 2021). This spillover infection might enable a sharp increase of confirmed cases in a total population, leading to a peak period and a high fatality rate. The viral transmission from a young group to an old-age group should be effectively managed by stringent quarantine.

In general, vaccination is prioritized for the elderly and younger age groups are subsequently vaccinated. The preventive effect of vaccination on the elderly can be compromised by high level of infection in younger groups who have yet to be immunized. Nevertheless, vaccination of younger age groups has a preventive effect on elderly groups as well. Thus, the sequential vaccination effect starting from the young to the elderly (at 3-month intervals) and vice versa should be evaluated based on the age-specific short-term incidence and mortality of SARS-CoV-2. Several variants appeared in an intermittent base period between the third and fourth peaks. The confirmed cases within the young age groups increased in association with the dominant virus strain; however, confirmed cases among the old age groups continued to decrease even before the effect of vaccination appeared (Figure 5D). The COVID-19 incidence during the intermittent base period was higher than in other base periods with obvious shifts in the infected age groups. Even with high levels of infection by the Delta variant in the young age group, the new variant showed delayed spread to the older population, although it was at an elevated base incidence during a long intermittent period (22 weeks, Figure 5D). Perhaps to spread the Delta variant from younger people to those in older groups, more confirmed cases are needed to cross the threshold that triggers spread to older persons. This suggests a difference between Delta and non-Delta variants.

RNA viruses, unlike DNA viruses, generate more mutations during replication due to higher error rates of their intrinsic polymerase (Holland et al., 1982). RNA virus yields a large group of genetically similar quasispecies (Domingo, 1998). Such mutation-prone expansion of zoonotic RNA virus provides the molecular basis for the acquisition of fitness to adapt to the human host. Based on our findings and studies by other groups, we further hypothesized that the directional selection of SARS-CoV-2 increases fitness to young age group with decreased fatality (Li et al., 2022; Shuai et al., 2022). In our results, fatality of SARS-CoV-2 decreased in Delta peak compared to previous three peaks with the main SARS-CoV-2-infected age group shifting to a younger group. Moreover, Delta virus infection showed delayed spread to older population. One study demonstrated a higher transmission in young age group during the third wave compared to previous first and second waves in Canada where vaccination was only limited to long term care residents and front line health care workers (McAlister et al., 2021). Other human studies showed reduced fatality and hospitalization rate in newer SARS-CoV-2 variants (Fan et al., 2021; Emani et al., 2022). Delta variants were less pathogenic compared to previous isolates including Alpha and Beta variants in a mouse model (Shuai et al., 2022). In addition, more frequent intra-host single-nucleotide SARS-CoV-2 variant accumulation was observed in young patients compared to old patients, suggesting higher chance for genetic adaptation in younger hosts (Li et al., 2022). Overall, these studies support our hypothesis about SARS-CoV-2 evolution despite the high social contact among young people and prior vaccination of old aged group still significantly contribute higher level of SARS-CoV-2 infection in younger age groups.

During the intermittent base period, South Korea successfully controlled the spread of COVID-19 and introduced a low-level quarantine policy for a 2- to 3-month period until confirmed cases sharply increased (Figure 2, Base 3). At the largest peak of confirmed cases (Delta peak 2), there were numerous deaths in the elderly despite a vaccination rate of more than 80%. Vaccines effectively protected at-risk groups in earlier stages of the pandemic when mutations were less frequent but were less effective in protecting against Delta variants (Pouwels et al., 2021). In South Korea, because stringent quarantines played a critical role in lowering the fatality rate as well as the number of confirmed cases, vaccines likely less contributed against infection of Delta variants compared to other countries with higher numbers of confirmed cases and deaths.

Israel introduced early vaccination unlike Sweden, which adopted a less stringent quarantine strategy accompanied by a vaccination program (Barda et al., 2021). Sweden subsequently had a lower prevalence of Delta variants than Israel (Paterlini, 2021). Therefore, the lower burden from Delta variants shown in Sweden may be ascribed to herd immunity achieved by lower levels of quarantine rather than to a vaccine effect. While some studies report rapid decline in SARS-CoV-2 specific neutralizing IgM and IgG levels (Xiang et al., 2021), other studies reported the long-lasting immunological memory against SARS-CoV-2 (Jung et al., 2021; Keeton et al., 2022; Tarke et al., 2022). Previous studies also suggested that natural infection induces a broader spectrum of protection against multiple variants (Dan et al., 2021; Gazit et al., 2021). In South Korea and Singapore, both of which had successful quarantines, the highly transmissible Delta variant spread during low-level quarantines despite high vaccination rates. A high proportion of the unvaccinated populations, especially in older age groups, were vulnerable to Delta variants. The Delta variant outbreak revealed an unexpected problem resulting from stringent quarantine policies and suggested the need for more sophisticated policy decisions.

For sex difference in SARS-CoV-2 fatality, recent studies have shown higher mortality of SARS-CoV-2 in men compared to women (Chanana et al., 2020; Jin et al., 2020; Sha et al., 2021). Another study also reported significant lower risk of in-hospital death among COVID-19 infected patients in women in South Korea (Her et al., 2022). However, in this study, there was no clear difference in sex-specific fatality rate with that of 0.90% for men and 0.92% for women. The discrepancy between these results is presumed to be due to the difference in whether the study subjects were hospitalized patients or total confirmed patients.

In summary, South Korea’s strict quarantine policy included isolation of infected persons and strict mask policy. The value of quarantines is measured by their effectiveness in early control of unexpected outbreaks and the level of protection they provide for high-risk groups. Since reducing effective contact in older individuals reduces mortality (Gondim and Machado, 2020; Tran Kiem et al., 2021), an age-dependent two-track quarantine is needed to maintain high-level protection for those aged 60 and older while keeping low-level measures in place for younger age groups to secure herd immunity. In this scenario, booster vaccinations may be prioritized for older persons while limiting vaccinations for younger age groups to once each winter. Knowing the true number of infection cases will aid in adjusting quarantine policy to reflect the rapid change of infection status of SARS-CoV-2 or newly emerging infectious diseases in the future. The epidemiologic data from the COVID-19 outbreak provided useful information for effective responses to the unfinished COVID-19 and possible future pandemics.

## Data Availability Statement

The original contributions presented in the study are included in the article/Supplementary Material, further inquiries can be directed to the corresponding author.

## Author Contributions

HC and SS performed the analysis and wrote the manuscript. S-US, S-JH, and M-GR designed the studies and wrote the manuscript. All authors contributed to the article and approved the submitted version.

## Conflict of Interest

The authors declare that the research was conducted in the absence of any commercial or financial relationships that could be construed as a potential conflict of interest.

## Publisher’s Note

All claims expressed in this article are solely those of the authors and do not necessarily represent those of their affiliated organizations, or those of the publisher, the editors and the reviewers. Any product that may be evaluated in this article, or claim that may be made by its manufacturer, is not guaranteed or endorsed by the publisher.

## Footnotes

1
http://ncov.mohw.go.kr/
