# Supplementary material for: High Level of SARS-CoV-2 Infection in Young Population Is a Predictor for Peak Incidence
Source: Front Microbiol. 2022 May 31;13:891646. doi: 10.3389/fmicb.2022.891646 (PMC9195141; doi:10.3389/fmicb.2022.891646)
Supplement: Supplementary file 1 [file Data_Sheet_1.PDF]

High level of SARS-CoV-2 infection in young population is a predictor for peak incidence

Haeyoun Choi, Sun Shin, Seung-Jin Hong, Sang-Uk Seo, and Mun-Gan Rhyu

Department of Microbiology, College of Medicine, The Catholic University of Korea, Seoul, Republic of Korea

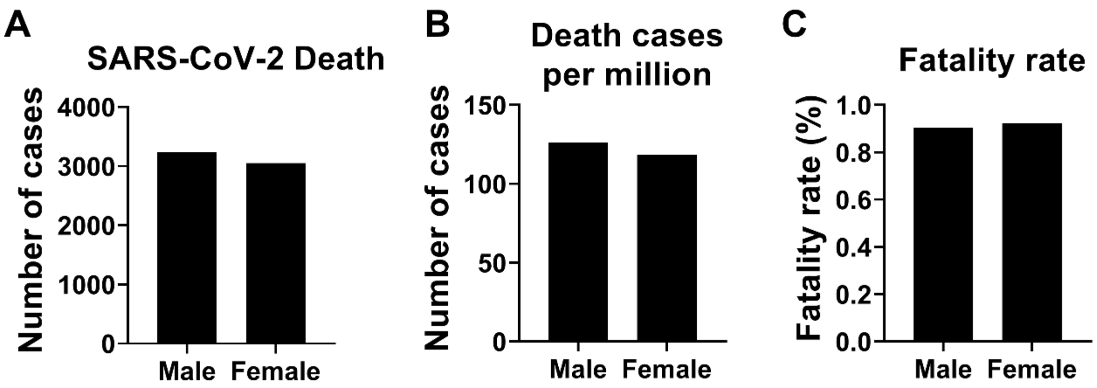

Supplementary Figure 1. Analysis of sex-specific mortality. Total SARS-CoV-2 death cases from January 2020 to January 15th, 2022 were analyzed. (**A-C**) Total number of deaths (**A**), death cases per million (**B**), and case fatality rate (**C**) are shown.

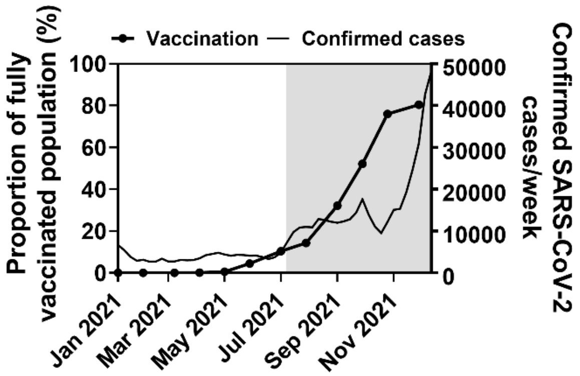

Supplementary Figure 2. Share of people who completed COVID-19 vaccination. Total number of people who were fully vaccinated were divided by the total population to calculate the proportion of fully vaccinated population in South Korea. Also, confirmed SARS-CoV-2 cases per week are shown. Delta peak period is shown in gray.
